# Cross-sectional study of dietary practices from the Dietary Guidelines for the Brazilian Population among users of a Basic Health Unit in Pelotas, 2022

**DOI:** 10.1590/S2237-96222025v34e20240420.en

**Published:** 2025-08-04

**Authors:** Mabel Nilson Alves, Denise Petrucci Gigante, Gicele Costa Mintem, Francine Silva dos Santos

**Affiliations:** 1 Universidade Federal de Pelotas, Faculdade de Nutrição, Programa de Pós- Graduação Nutrição e Alimentos, Pelotas, RS, Brazil; 2 Universidade Federal de Ciências da Saúde de Porto Alegre, Departamento de Nutrição, Porto Alegre, RS, Brazil

**Keywords:** Healthy diet, Primary healthcare, Dietary Guidelines, Public health, Cross-sectional studies, Dieta saludable, Atención Primaria de Salud, Guías Alimentarias, Salud Pública, Estudios Transversales

## Abstract

**Objective:**

Describe dietary practices recommended in the Dietary Guidelines and the association with sociodemographic factors and lifestyle habits in users of the nutrition service at a Basic Health Unit in Pelotas, Rio Grande do Sul in 2022.

**Methods:**

Cross-sectional study with individuals aged ≥20 years who received nutritional care. The questionnaire used included a scale validated for the Brazilian population, which considers three domains of food consumption: domestic planning and organization, food choice and eating habits. Practices with a frequency of 60% or more were considered appropriate to the recommendations of the Dietary Guidelines.

**Results:**

Among the 162 interviewees, only the eating practices related to the domain of eating habits were adequate. The mean adequacy score was 39.0 points with a standard deviation of 5.4 points and a median of 39.0 points. The mean adequacy score was statistically higher among respondents who declared themselves to be race/skin color brown/black, who had 40.4 points with a 95% confidence interval (95%CI 39.1; 41.7) and among those active in their free time, who had 40.4 points (95%CI 39.0; 41.8), after adjustments for demographic and socioeconomic variables.

**Conclusion:**

The adequacy score values were higher in brown/black individuals and those who practiced some kind of physical activity during leisure time. Practices related to the domains of planning and food choices should be prioritized in nutritional counseling.

## Resumen

**Objetivo:**

Describir las prácticas alimentarias recomendadas en la Guía Alimentaria y la asociación con factores sociodemográficos y hábitos de vida en usuarios del servicio de nutrición de una Unidad Básica de Salud de Pelotas, Rio Grande do Sul en 2022.

**Métodos:**

Estudio transversal con individuos ≥20 años que recibieron atención nutricional. El cuestionario utilizado incluyó una escala validada para la población brasileña, que considera tres dominios del consumo de alimentos: planificación y organización doméstica, elección de alimentos y hábitos alimentarios. Las prácticas que obtuvieron una frecuencia de 60% o más se consideraron apropiadas a las recomendaciones de la Guía de Alimentos.

**Resultados:**

Entre los 162 entrevistados, sólo las prácticas alimentarias relacionadas con el dominio de los hábitos alimentarios resultaron adecuadas. La puntuación media de adecuación fue de 39,0 puntos con una desviación estándar de 5,4 puntos y una mediana de 39,0 puntos. El puntaje promedio de adecuación fue estadísticamente mayor entre los encuestados que autodeclararon su raza/color de piel como marrón/negro, quienes presentaron 40,4 puntos con un intervalo de confianza del 95% (IC95% 39,1; 41,7) y entre aquellos activos en el ocio, quienes presentaron 40,4 puntos (IC95% 39,0; 41,8), después de los ajustes por variables demográficas y socioeconómicas.

**Conclusión:**

Los valores del puntaje de adecuación fueron mayores en los individuos de piel morena/negra y en aquellos que practicaban actividad física en su tiempo libre. Las prácticas relacionadas con los dominios de la planificación y la elección de alimentos deben priorizarse en el asesoramiento nutricional.

**Ethical aspects**

This research respected ethical principles, having obtained the following approval data:

Research Ethics Committee: Universidade Federal de Pelotas

Opinion number: 5.373.094

Approval date: 27/4/2022

Certificate of Submission for Ethical Appraisal: 57491222.2.0000.5316

Informed Consent Form: Obtained from all participants prior to collection.

## Introduction

The National Primary Care Policy comprises a vast set of health actions that must be developed through integrated care practices, qualified management and must target the population in a defined territory (1). From this perspective, the National Food and Nutrition Policy aims to improve the eating, nutrition and health conditions of the Brazilian population, through the promotion of adequate and healthy eating practices, food and nutrition surveillance, prevention and comprehensive care of food and nutrition-related illnesses (2). 

For these actions, the Ministry of Health advises the Primary Healthcare professionals to use the Dietary Guidelines for the Brazilian Population (3). In 2014, it was updated and included the NOVA classification, based on the extent and purpose of the degree of food processing (3). It expanded the recommendations beyond the biological dimension, by considering environmental, economic, social, cultural aspects, and food as a right for all. It is a pioneering instrument in terms of encouraging the development of culinary skills, the transmission of culinary knowledge between generations, of encouraging sharing tasks in the kitchen and eating as a family (4).

In contrast to what is recommended by the Dietary Guidelines, we note that in 16 years Brazilians reduced their consumption of natural foods by 7.0%, increased their consumption of ultra-processed foods by 46.0% and increased their purchase of ready-made frozen meals by 250.0% (5). In the same period, the prevalence of chronic diseases related to poor diet increased (5). In Primary Healthcare, 37.0% of adult individuals reported eating hamburgers and/or sausages the day before and 54.0% drank sweetened beverages in the same period (6).

Given the obvious importance of the Dietary Guidelines further investigation into adherence to their best practices in the Primary Healthcare is necessary. Study eating practices based on Dietary Guidelines for the Brazilian Population of individuals assisted in the Primary Healthcare, especially those already treated by a nutritionist, provides a better understanding for its implementation in this population, allowing for better health outcomes (7-8). Therefore, the aim of this study was to describe dietary practices recommended in the Dietary Guidelines for the Brazilian Population and the association with sociodemographic factors and lifestyle habits in users of the nutrition service at a Basic Health Unit in Pelotas, Rio Grande do Sul, in 2022.

## Methods

### Study design 

Cross-sectional study with selection of the target population by convenience.

### Context

The study was carried out in a Basic Health Unit located in the Três Vendas neighborhood of the municipality of Pelotas, Rio Grande do Sul. Pelotas has 325,685 residents and a Municipal Human Development Index of 0.739 (9). The municipality’s Primary Healthcare is organized into Healthcare Networks, which perform coordinated health actions and services in a complementary manner and on a territorial basis, with the Basic Health Units being the first stop for users in the Networks. Currently, Pelotas has 50 Basic Health Units (10). Of these, three Units are linked to the Federal University of Pelotas where teaching, research and extension activities are developed (11). The Basic Health Unit, where this study was conducted, was chosen by convenience among these, as it is the workplace of one of the authors, to diagnose the adequacy of the eating habits of patients treated by the nutrition service. At the time of data collection, this Unit had a registered population of 3,500 people and presented a mixed care model, that is, a traditional Basic Health Unit and Family Health Strategy. In its assigned area, it only has one farmers fair, that sells its products only one day a week.

### Participants

The target population of this study consists of individuals aged 20 years or older who received nutritional care. The selection of individuals was through analysis of all records of patients treated by the Unit’s nutrition team between 2015 and 2019. As an inclusion criterion, we added the requirement to reside in the area covered by the Basic Health Unit, at the time of nutritional care and data collection for the study. As an exclusion criterion, any condition (physical or mental) that limited the response of those selected to the questionnaire was considered. 

### Variables

The outcomes in the present study were dietary practices that followed the recommendations of the Dietary Guidelines for the Brazilian Population. For this purpose, the adequacy score of the scale validated for the Brazilian population was used, consisting of 24 questions and divided into three domains. The domain of domestic planning and organization contains questions about seven practices; the domain of “eating habits” contains eight practices; and the domain of “food choices” contains nine practices. Each question can be answered with never, rarely, often, and always. For each answer there is a score that, when added together, results in the adequacy score that varies from a minimum score of 0 to a maximum of 72 points, with a value of ≥42 points being considered adequate (12). In addition, the frequency parameter of ≥60% was used for each practice to classify it as adequate frequency (13). In the present study, the scale was modified from affirmative to interrogative form and from the first to the third person. 

The explanatory variables were demographic, socioeconomic and lifestyle. The demographics analyzed were: sex (female or male), age in years and analyzed in two categories, adults (20 to 59 years) and elderly (≥60 years), and self-declared race/skin color according to the categories established in national studies (14) and grouped later into brown/black or white. 

As socioeconomic variables, the study considered: level of education (year and study level completed by the interviewee and converted as a discrete quantitative variable in years of study) analyzed in dichotomous form (incomplete elementary school: up to 7 years of study and complete elementary school: 8 years and more) and per capita family income, analyzed in tertiles, and obtained by adding all the amounts received by the participant and by persons living in the same house, divided by the number of residents in the household.

Lifestyle variables: for physical activity, we used the question “Do you do any sports, exercise or physical activity in your leisure or free time?” with yes or no answer options and analyzed in this way. For smoking, anyone who smokes is considered a smoker regardless of the number of cigarettes, frequency and duration of the smoking habit (14). Initially, this was presented like three categories: smoker, ex-smoker or never smoked, and in the bivariate analyses and linear regression, the variable was presented in a dichotomous form as “yes” and “no” for smoking habit, the latter being the category that included non-smokers and ex-smokers.

### Data collection

Eligibility verification and data collection took place from June to November 2022, by trained interviewers (Nutrition students). Data collection was carried out in physical and electronic medical records and in interviews via telephone or in person. 

The questionnaire was built using Epicollect5 software. As measures for data quality control, a pilot study was conducted to adapt the questionnaire and study logistics, the interviewers were previously trained and received an instruction manual for field work. The manual contained guidelines on how to correctly complete the questionnaire and use the software. Furthermore, the study coordinating team was composed of nutritionists with extensive experience in Primary Healthcare and in conducting epidemiological studies. 

### Bias

The design of the present study does not allow establishing a causal relationship; therefore, it describes and shows associations between the outcomes and the explanatory variables without indicating possible causes. As it is a convenience sample, it presents restricted generalization, and it is not possible to extrapolate the results to the entire population of users of Primary Healthcare.

### Study size

From 2015 to 2019, the number of nutrition consultations was 2850, 1103 of which were from the Unit’s assigned area. Of these, 380 users were under 20 years of age and 487 visited the Basic Health Unit only to have anthropometric measurements taken for the Bolsa Família Program, not for nutritional care. Thus, 236 individuals were eligible for the study. After efforts to contact these individuals, the following were identified: 15 changes of address, 14 deaths and one individual was unable to respond to the questionnaire. This yielded a total of 206 individuals eligible for the study. Of these, 162 were interviewed (78.6%), refusals represented 13.1% and losses 8.3%.

### Statistical methods

The data obtained in the spreadsheet generated by Epicollect5 were imported by the statistical program Stata 16.1 (Stata Corporation, College Station, Texas, United States of America). Initially, the frequency distributions of dichotomous, ordinal and categorical variables were observed regarding the normality of the distribution. Afterwards, the frequency of all variables of interest, description of numerical variables in means and standard deviation and proportion for categorical variables were checked. The level of statistical significance was p-value less than 5.0%.

The prevalence of the variables and their 95% confidence intervals (95%CI) were calculated. Bivariate analyses were conducted between the outcomes and explanatory variables using Chi-square and Fisher’s exact statistical tests. Linear regression was conducted to evaluate the means of the adequacy scores in relation to the explanatory variables using the Wald test. For the adjusted analyses, a hierarchical model in levels was used, with the first level composed of demographic variables (sex, age and race/skin color); the second level, by socioeconomic variables (education and per capita income) and the third level, by lifestyle variables (smoking and physical activity). Estimates were adjusted for variables at the same level and the previous level, considered as possible confounding factors, and those that presented a p-value lower than 20.0% at their hierarchical level remained in the model (15). 

## Results

Of the 162 interviews conducted, 57.4% were in person. Women represented 78.8%; people aged 40 or over, 83.3%; self-declared as race/skin color white, 57.4%; 62.7% reported having less than eight years of schooling and 65.2% did not practice any physical activity during leisure time (Table 1).

**Table 1:** Absolute and relative distribution of users of a nutrition service in Primary Healthcare, according to sociodemographic and lifestyle variables. Pelotas, 2022 (n=162)

Variables	n (%)
Total	162 (100)
Gender	
Male	36 (22.2)
Female	126 (77.8)
**Age** (years)^a^	
20-39	27 (16.7)
40-59	71 (43.8)
≥60	64 (39.5)
**Race/skin color**	
White	93 (57.4)
Brown	23 (14.2)
Black	46 (28.4)
**Education** (years)^a^	
0-4	100 (62.1)
5-7	1 (0.6)
8-10	11 (6.8)
11 or more	49 (30.5)
**Per capita income** (reais)^b^	
1st tertile (0-454.5)	54 (33.8)
2nd tertile (442.5-954.0)	53 (33.1)
3rd tertile (954.1-4,893.3)	53 (33.1)
Smoking^a^	
Never smoked	87 (54.0)
Ex-smoker	47 (29.2)
Smoker	27 (16.8)
Physical activity during leisure time^a^	
No	105 (65.2)
Yes	56 (34.8)

^a^n=161; ^b^n=160

None of the participants complied with any practice in the domestic planning and organization domain. In the “eating habits” domain, there was adequacy in seven of the eight practices, with a range of adequacy frequencies from 72.8% to 95.7%. The most appropriate practice in this domain was never or rarely eating meals at the work or study table. In the “food choices” domain, there was adequacy in five of the nine related practices with a frequency range of adequacy from 60.5% to 66.0%. The most appropriate practice in this domain was never or rarely eating sandwiches, savory snacks, or pizzas instead of food (Table 2).

**Table 2:** Frequencies and 95% confidence intervals (95%CI) of dietary practices of users of nutrition services in Primary Healthcare. Pelotas, 2022 (n=162)

Domain and Scale Component	Never/rarely	Often/always
	% (IC95%)	% (IC95%)
**Domestic planning and organization**		
Eats fruit or nuts in small snacks	52.5 (45.0; 60.1)	47.5 (40.0; 55.3)
Chooses locally produced fruits, vegetables and legumes	39.9 (38.4; 41.3)	52.5 (44.7; 60.1)
Takes some food in case they feel hungry later	76.5 (69.4; 82.5)	23.5 (17.5; 30.7)
Plans meals	69.7 (62.2; 76.4)	30.5 (23.6; 37.8)
Varies the consumption of beans with other legumes	54.9 (47.2; 62.5)	45.1 (37.5; 52.8)
Uses whole-wheat flour frequently	77.8 (70.7; 83.6)	22.2 (16.4; 29.3)
Usually eats fruit for breakfast	80.2 (73.3; 85.7)	19.8 (14.3; 26.7)
**Eating habits**		
Usually eats meals sitting at the table	26.5 (20.3; 33.9)	73.5 (66.1; 79.7)
Takes time to eat meals without rushing	24.1 (18.1; 31.3)	75.9 (68.7; 81.9)
Usually participates in meal preparation	27.2 (20.8; 34.6)	72.8 (65.4; 79.2)
Residents share the tasks involved in preparing and eating meals at home	48.8 (41.1; 56.5)	51.2 (43.5; 58.9)
Uses meal times to resolve other issues and ends up not eating	82.7 (76.5; 87.8)	17.3 (12.2; 23.9)
Usually eats meals at the work or study table	95.7 (91.2; 97.9)	4.3 (2.1; 8.8)
Usually eats meals sitting on the sofa or in bed	74.1 (66.7; 80.3)	25.9 (19.7; 33.3)
Usually skips at least one of the main meals	72.8 (65.4; 79.2)	27.2 (20.8; 34.6)
**Food choices**		
Gives preference to organic fruits, vegetables and legumes	35.8 (28.8; 43.5)	64.2 (56.5; 71.3)
Usually buys food at farmers or street markets	66.7 (59.0; 73;5)	33.3 (26.5; 41.0)
Usually eats candies, chocolates, and other treats	40.7 (33.4; 48.5)	59.3 (51.5; 66.6)
Usually drinks industrialized juices	62.9 (55.2; 70.1)	37.0 (29.9; 44.8)
Usually goes to fast-food restaurants or snack bars	60.5 (52.7; 67.8)	39.5 (32.2; 47.3)
Eats snacks between meals frequently	63.0 (55.2; 70.1)	37.0 (29.9; 44.8)
Usually drinks soda	45.7 (38.1; 53.5)	54.3 (46.6; 61.9)
Usually exchanges food for sandwiches, snacks, pizzas	66.0 (58.4; 73.0)	33.9 (27.0; 41.6)
Usually adds sugar when drinking coffee or tea	46.6 (39.0; 54.4)	53.4 (45.6; 61.0)

Brown/black individuals, compared to white individuals, reported more frequently “often” and “always” choosing locally produced fruits and vegetables (59.4% vs. 47.3%; p-value 0.003); always eating fruit for breakfast (13.0% vs. 4.3%; p-value 0.036); never use mealtimes to resolve other things and end up not eating (21.5 vs. 9.7%; p-value 0.002); never eating meals on the couch or in bed (29.0% vs. 16.1%; p-value 0.037) and never eating sandwiches, savory snacks or pizza instead of food for lunch or dinner (17.4% vs. 3.2%; p-value 0.021). Brown/black individuals, compared to white individuals, reported always skipping main meals (lunch or dinner) less frequently (4.4% vs. 8.6%; p-value 0.049) (Supplementary Table 3). Individuals who practiced physical activity, compared to those who did not, reported always eating fruit for breakfast more frequently (12.5% vs. 5.7%; p-value 0.043) and always drinking soft drinks less frequently (5.4% vs. 11.4%; p-value 0.012) (Supplementary Table 6). Association of eating practices with other explanatory variables such as biological sex, age group, education and smoking are presented in Supplementary Tables 1, 2, 4 and 5.

**Table 3:** Unadjusted and adjusted means and 95% confidence interval (95%CI) of the adequacy score by study variables in users of nutrition services in Primary Healthcare. Pelotas, 2022 (N=162)

Variables	Unadjusted (95% CI)	p-value^a^	Adjusted (IC95%)	p-value^a^
**1st level: demographic variables** (n=162)				
Gender		0.601		0.872
Female	40.1 (38.1; 42.2)		39.0 (38.0; 40.0)	
Male	38.6 (36.8; 40.4)		39.2 (37.3; 41.0)	
**Age in complete years**		0.746		0.498
Adults	38.9 (37.8; 40.0)		39.9 (38.4; 41.3)	
Elderly	39.2 (37.9; 40.6)		39.1 (38.1; 40.7)	
**Race/skin color**		0.007		0.007
White	39.1 (38.1; 40.7)		39.1 (38.1;40.7)	
Brown/black	39.1 (38.1; 40.7)		39.1 (38.1; 40.7)	
**2nd level: socioeconomic variables** (n=161/160)				
Education		0.335		0.404
Incomplete elementary school	39.3 (38.2; 40.4)		39.3 (38.2; 40.4)	
Completed elementary education	38.5 (37.1; 39.8)		39.9 (38.4; 41.3)	
**Per capita income at home**		0.325		0.256
1st tertile	39.9 (38.4; 41.3)		39.8 (38.3; 41.2)	
2nd tertile	38.3 (36.8; 39.8)		38.1 (36.6; 39.5)	
3rd tertile	39.0 (37.6; 40.5)		39.2 (37.8; 40.7)	
**3rd level: behavioral variables** (n=161)				
Smoking		0.255		0.379
No	38.8 (37.9; 39.8)		38.9 (38.0; 39.8)	
Yes	40.1 (38.1; 42.2)		39.9 (38.4; 41.3)	
**Physical activity**		0.021		0.023
No	38.3 (37.3; 39.4)		38.4 (37.3; 39.4)	
Yes	40.4 (39.0; 41.8)		40.4 (39.0; 41.8)	

^a^Wald test; adjustment was performed for variables of the same level and the previous level with p-valor<0.20.

The mean adequacy score was 39.0 points with a standard deviation of 5.4 points and a median of 39.0 points. The average was statistically higher among respondents who declared themselves to be brown/black, at 40.4 points with a 95% confidence interval (95%CI 39.1; 41.7) and among those active during their free time, who had 40.4 points (95%CI 39.0; 41.8), even after adjustments for the variables included in the first and second levels of the analysis model. Respondents who declared themselves as brown/black had an average score of 2.44 points, higher than the average score of white respondents. Those who practiced any physical activity had an average score of 2.00 points higher than those who did not practice a physical activity (Table 3). 

## Discussion

The findings of the present study show that adults and elderly individuals who received nutritional care at a basic health unit did not follow completely any of the food practices recommended in the Dietary Guidelines for the Brazilian Population for the domain of domestic planning and organization, while in the domain of “eating habits” there was adequacy in most practices and in five of the nine practices in the domain of “food choices”. Furthermore, the mean score on the scale did not reach the score considered for adequacy (≥42 points) (12), being higher among interviewees who declared themselves to be brown/black in relation to whites and among those who practiced physical activity compared to those who did not. 

The lack of adequacy of practices related to domestic planning and organization may reflect the difficulty to understand the practices in this domain, given that the percentage of individuals with incomplete elementary education identified in the study was higher than the national prevalence (16). Added to this is the financial condition of the individuals, who had a monthly per capita income much lower than that identified at national and regional levels in the same year (17). 

Food choices are influenced by economic factors, in addition to social and political factors, which determine the food situation of the population, communities and individuals (18). This fact was evidenced in the population studied, since there was a higher prevalence of never or rarely buying food at farmers market, and a higher prevalence of often or always consuming sweets and soft drinks. This relationship corroborates the increase in the price of natural or minimally processed foods and the reduction in the price of ultra-processed foods that occurred in the last three decades (19). Another factor is that 80.0% of farmers markets in the city of Pelotas, Rio Grande do Sul, are concentrated in the central region, with few options for residents of the neighborhoods where the individuals in this study live (20). The results of this study related to the lower adequacy in the domains of domestic planning and organization and food choices highlight the need for measures to ensure a healthy and sustainable food system to contribute to the improvement of dietary practices related to these domains (21). The intensified work of Primary Healthcare teams through educational campaigns on healthy eating practices, combined with the municipal government’s actions to increase the number of family farming fairs, with an increase in demand for these products, could promote greater diversity and access to healthy foods. Still, government financial subsidies are needed to modulate the food system to promote adequate and healthy eating among the individuals studied. 

In turn, the practices in the eating habits domain showed a higher prevalence of compliance with the recommendations of the Dietary Guidelines. It is clear that they are related to eating behavior and, therefore, do not depend on financial conditions for their implementation. Furthermore, they represent habits still maintained by these individuals, exemplifying an intangible Brazilian heritage that needs to be preserved and perpetuated (22). The Primary Healthcare team has the potential to disseminate and encourage these practices through the family health strategy (1).

The mean and median of the adequacy score are below the cutoff point considered for those who have adequate and healthy eating practices (≥42 points) (12), reinforcing that these practices need to improve in this population. However, it was still higher than that identified in workers in the food service trade (snack bars, restaurants, bakeries, coffee shops, supermarkets and hypermarkets) in Fortaleza, Ceará, whose average adequacy score was 35 points (23) and higher than the average adequacy score of 36 points found in a study carried out with 900 adults from all over Brazil (9). The higher mean adequacy score found in this study compared to others reported previously may be the result of the nutritional treatment received at the Basic Health Unit, exemplifying that nutritional interventions can influence the quality of food even in low-income individuals (24). 

Individuals who self-identified as brown/black had the highest mean scores for adequacy of dietary practices in line with the recommendations of the Dietary Guidelines for the Brazilian Population, which is related to reports about often or always choosing locally produced fruits and vegetables, never or rarely skipping meals and never swapping lunch or dinner for sandwiches, savory snacks or pizza. These findings may be due to the traditional Brazilian diet still maintained among brown/black individuals (25). At a national level, a higher consumption of natural and minimally processed foods, especially rice and beans, was identified among brown/black individuals (26).

The mean score for the adequacy of dietary practices in accordance with the recommendations of Dietary Guidelines for the Brazilian Population was also higher among those who practiced physical activity during leisure time, which is related to the greater frequency of always consuming fruit for breakfast and never or rarely consuming soft drinks. This is in line with the Health Belief Model, which suggests that individuals tend to make healthy lifestyle choices to prevent/treat disease when they are aware that their actions have an impact on their health (27). 

The limitations of the study correspond to losses and refusals that represented 21.0% of eligible individuals. To minimize losses, when telephone contact was not successful, at least three attempts in different shifts, in-person searches were conducted at the address of the individuals in four attempts on different days and shifts. Contact with neighbors to check the possibility of a change of address or death was also carried out. The national registry of deceased people was also consulted. On the other hand, the number of losses may have been overestimated given that it was not possible to confirm whether all losses were in fact individuals eligible for the study, that is, residents of the area covered by the Basic Health Unit.

The present study contributes to scientific knowledge, by presenting the frequency of dietary practices recommended by the Dietary Guidelines for the Brazilian Population by individuals treated in Primary Healthcare who had already received nutritional treatment. It is worth mentioning that the instrument used to assess the outcome is also a differential of this study, since in addition to being a scale validated for the Brazilian population (28), it is an official instrument for assessing the diet included in the Meu SUS Digital application and in the Ministry of Health folder to assess adherence to the recommendations of the Dietary Guidelines for the Brazilian Population (29). Furthermore, as a scientific contribution, the present study analyzed each dietary practice in relation to the variables of sex, age, race/skin color, education, per capita income, smoking and physical activity, which broadens the understanding for planning actions aimed at improving the diet of individuals in Primary Healthcare. 

Therefore, in the present study, the mean adequacy score for compliance with practices recommended by the Dietary Guidelines was below the recommended value, with individuals who self-declared as brown/black and those who practiced physical activity showing higher averages in relation to those who self-declared as white and did not practice any physical activity. Individuals presented more difficulty in adapting eating practices related to the dimensions of domestic planning and organization and food choice. It is suggested that Primary Healthcare professionals continue and intensify their work in synergy with government actions to publicize, encourage and support healthy eating practices recommended by the Dietary Guidelines for the Brazilian Population.

## Data Availability

The database and analysis codes used in the research are available upon request to the authors.
