# Supplementary material for: Cross-sectional study of dietary practices from the Dietary Guidelines for the Brazilian Population among users of a Basic Health Unit in Pelotas, 2022
Source: Epidemiol Serv Saude. 2025 Aug 4;34:e20240420. doi: 10.1590/S2237-96222025v34e20240420.en (PMC12334227; doi:10.1590/S2237-96222025v34e20240420.en)
Supplement: Supplementary file 1 [file 2237-9622-ress-34-e20240420-suppl01.pdf]

Tabela Suplementar 1. Prevalências (%) de práticas alimentares conforme sexo de usuários do serviço de nutrição na Atenção Primária. Pelotas, Rio Grande do Sul, 2022 (N=162)

	Masculino				Feminino				
**Domínio e Componente da escala**	**Nunca**	**Raramente**	**Muitas Vezes**	**Sempre**	**Nunca**	**Raramente**	**Muitas Vezes**	**Sempre**	**p-valor[a]**
	**%**	**%**	**%**	**%**	**%**	**%**	**%**	**%**	
Planejamento e organização doméstica									
Come frutas ou castanhas em pequenos lanches	19,4	19,4	41,8	19,4	25,4	31,0	23,0	20,6	0,173
Escolhe frutas, verduras e legumes de produção local	22,2	8,3	27,8	41,7	31,8	20,6	23,8	23,8	0,090
Leva algum alimento para caso sentir fome	6,4	70,6	9,5	13,5	8,3	66,7	11,1	13,9	0,904
Planeja refeições	27,8	47,2	11,1	13,9	21,5	46,8	11,9	19,8	0,795
Varia o consumo de feijão com outras leguminosas	25,1	33,3	33,3	8,3	22,2	31,8	34,1	11,9	0,957
É comum usar farinha integral	8,3	75,0	11,1	5,6	6,4	69,8	15,1	8,7	0,848
Costuma comer fruta no café da manhã	5,6	77,8	13,9	2,7	7,1	72,2	11,1	9,5	0,639
Modos de comer									
Costuma fazer as refeições sentado(a) à mesa	22,2	8,3	2,8	66,7	15,9	9,5	14,3	60,3	0,237
Realiza as refeições com calma	22,2	0,0	13,9	63,9	16,7	7,9	19,0	56,4	0,269
Costuma participar do preparo das refeições	25,0	33,3	19,5	22,2	15,0	3,2	5,6	76,2	<0,001
Na casa as pessoas compartilham as tarefas que envolvem o preparo e consumo das refeições	27,8	25,0	13,9	33,3	17,5	30,2	8,7	43,6	0,337
Aproveita o horário das refeições para resolver outras coisas e acaba deixando de comer	5,6	80,6	8,3	5,6	17,5	64,3	12,7	5,6	0,233
Costuma fazer as refeições na mesa de trabalho ou estudo	8,3	88,9	2,8	0,0	7,9	87,3	3,2	1,6	1,000
Costuma fazer as refeições sentado(a) no sofá ou na cama	25,0	55,5	16,7	2,8	20,6	51,6	14,3	13,5	0,325
Costuma pular pelo menos uma das refeições principais	27,8	47,2	22,2	2,8	36,6	35,7	19,8	7,9	0,475
Escolhas alimentares									
Dá preferência pra frutas, verduras e legumes orgânicos	19,4	25,0	38,9	16,7	14,3	19,0	49,2	17,5	0,656
Costuma comprar alimentos em feiras livres ou de rua	13,9	44,4	36,1	5,6	19,8	49,2	17,5	13,5	0,105
Costuma comer balas, chocolates e outras guloseimas	13,9	27,8	55,6	2,7	20,6	19,8	44,5	15,1	0,116
Costuma beber sucos industrializados	19,4	44,5	25,0	11,1	15,9	46,8	26,2	11,1	0,960
Costuma frequentar restaurantes fast-foods ou lanchonetes	2,8	52,8	44,4	0,0	4,8	57,1	33,3	4,8	0,503
Tem o hábito de beliscar entre as refeições	19,4	44,5	22,2	13,9	19,8	42,9	22,2	15,1	1,000
Costuma beber refrigerante	19,4	36,2	36,1	8,3	9,5	33,3	47,6	9,6	0,342
Costuma trocar comida por sanduíches, salgados, pizzas	8,3	50,0	38,9	2,8	9,5	58,7	27,0	4,8	0,625
Quando bebe café ou chá, costuma colocar açúcar	8,4	44,5	13,8	33,3	8,8	36,0	9,6	45,6	0,541

[a]Qui-quadrado e exato de Fisher

Tabela Suplementar 2. Prevalências (%) de práticas alimentares conforme idade de usuários do serviço de nutrição na Atenção Primária. Pelotas, Rio Grande do Sul, 2022 (N=160)

Domínio e Componente da escala	Adultos (20 a 59 anos)				Idosos (≥60 anos)				
	Nunca	Raramente	Muitas Vezes	Sempre	Nunca	Raramente	Muitas Vezes	Sempre	p-valor[a]
	%	%	%	%	%	%	%	%	
Planejamento e organização doméstica									
Come frutas ou castanhas em pequenos lanches	26,0	26,0	31,3	16,7	21,9	32,8	18,7	26,6	0,175
Escolhe frutas, verduras e legumes de produção local	31,3	19,8	22,9	26,0	26,6	15,6	28,1	29,7	0,753
Leva algum alimento para caso sentir fome	6,3	66,7	11,5	15,5	7,8	75,0	6,3	10,9	0,551
Planeja refeições	15,6	53,1	12,5	18,8	32,8	37,5	10,9	18,8	0,069
Varia o consumo de feijão com outras leguminosas	19,8	33,3	34,4	12,5	28,1	29,7	32,8	9,4	0,654
É comum usar farinha integral	6,3	69,8	13,5	10,4	7,8	73,4	14,1	4,7	0,631
Costuma comer fruta no café da manhã	4,2	71,9	13,5	10,4	10,9	75,0	9,4	4,7	0,213
Modos de comer									
Costuma fazer as refeições sentado(a) à mesa	19,8	10,4	12,5	57,3	14,1	7,8	7,8	70,3	0,460
Realiza as refeições com calma	19,8	9,4	22,9	47,9	15,6	1,6	9,4	73,4	0,005
Costuma participar do preparo das refeições	19,8	5,2	9,4	65,6	14,1	19,2	7,8	60,9	0,099
Na casa as pessoas compartilham as tarefas que envolvem o preparo e consumo das refeições	19,8	29,2	7,3	43,7	20,3	28,1	14,1	37,5	0,543
Aproveita o horário das refeições para resolver outras coisas e acaba deixando de comer	19,8	60,4	14,6	5,2	6,3	79,7	7,8	6,2	0,031
Costuma fazer as refeições na mesa de trabalho ou estudo	11,5	84,4	3,1	1,0	3,1	92,2	3,1	1,6	0,255
Costuma fazer as refeições sentado(a) no sofá ou na cama	21,9	52,1	13,5	12,5	18,8	54,7	17,2	9,4	0,832
Costuma pular pelo menos uma das refeições principais	36,5	32,3	26,0	5,2	31,3	46,9	12,5	9,4	0,074
Escolhas alimentares									
Dá preferência pra frutas, verduras e legumes orgânicos	17,7	20,8	44,8	16,7	12,5	18,8	50,0	18,7	0,795
Costuma comprar alimentos em feiras livres ou de rua	17,7	45,8	22,9	13,6	20,3	51,6	20,3	7,8	0,671
Costuma comer balas, chocolates e outras guloseimas	19,8	19,8	47,9	12,5	17,2	25,0	45,3	12,5	0,886
Costuma beber sucos industrializados	21,9	41,7	23,9	12,5	9,4	54,6	26,6	9,4	0,143
Costuma frequentar restaurantes fast-foods ou lanchonetes	7,2	50,0	36,5	6,3	0,0	65,6	34,4	0,0	0,011
Tem o hábito de beliscar entre as refeições	22,9	39,6	20,8	16,7	14,1	48,4	25,0	12,5	0,405
Costuma beber refrigerante	13,5	29,2	44,8	12,5	9,4	42,1	43,8	4,7	0,186
Costuma trocar comida por sanduíches, salgados, pizzas	10,4	47,9	35,4	6,3	7,8	68,8	21,8	1,6	0,060
Quando bebe café ou chá, costuma colocar açúcar	9,4	30,2	9,4	51,0	7,9	49,2	11,1	31,8	0,066

[a]Qui-quadrado e exato de Fisher

Tabela Suplementar 3. Prevalências (%) de práticas alimentares conforme raça/cor da pele de usuários do serviço de nutrição na Atenção Primária. Pelotas, Rio Grande do Sul, 2022 (N=162)

Domínio e Componente da escala	Pardos/pretos				Brancos				
	Nunca	Raramente	Muitas Vezes	Sempre	Nunca	Raramente	Muitas Vezes	Sempre	p-valor[a]
	%	%	%	%	%	%	%	%	
Planejamento e organização doméstica									
Come frutas ou castanhas em pequenos lanches	26,1	24,6	23,2	26,1	22,6	31,2	30,1	16,1	0,333
Escolhe frutas, verduras e legumes de produção local	20,3	20,3	37,7	21,7	36,6	16,1	15,0	32,3	0,003
Leva algum alimento para caso sentir fome	5,8	71,0	5,8	17,4	7,5	68,8	12,9	10,8	0,320
Planeja refeições	22,5	49,5	10,8	17,2	23,2	43,5	13,0	20,3	0,878
Varia o consumo de feijão com outras leguminosas	21,7	31,9	31,9	14,5	23,7	32,3	35,5	8,6	0,706
É comum usar farinha integral	5,8	72,5	10,1	11,6	7,5	69,9	17,2	5,4	0,332
Costuma comer fruta no café da manhã	2,9	76,8	7,3	13,0	9,7	71,0	15,0	4,3	0,036
Modos de comer									
Costuma fazer as refeições sentado(a) à mesa	17,4	14,5	13,0	55,1	17,2	5,4	10,7	66,7	0,209
Realiza as refeições com calma	8,7	7,3	20,3	63,7	24,7	5,4	16,1	53,8	0,062
Costuma participar do preparo das refeições	20,3	5,8	8,7	65,2	15,1	12,9	8,6	63,4	0,461
Na casa as pessoas compartilham as tarefas que envolvem o preparo e consumo das refeições	18,8	31,9	7,3	42,0	20,4	26,9	11,8	40,9	0,768
Aproveita o horário das refeições para resolver outras coisas e acaba deixando de comer	21,7	56,5	10,2	11,6	9,7	76,3	12,9	1,1	0,002
Costuma fazer as refeições na mesa de trabalho ou estudo	11,5	85,5	1,5	1,5	5,4	89,3	4,3	1,0	0,403
Costuma fazer as refeições sentado(a) no sofá ou na cama	29,0	44,9	10,1	15,9	16,1	58,1	18,3	7,5	0,037
Costuma pular pelo menos uma das refeições principais	46,4	30,4	18,8	4,4	25,8	44,1	21,5	8,6	0,049
Escolhas alimentares									
Dá preferência pra frutas, verduras e legumes orgânicos	13,0	17,4	49,3	20,3	17,2	22,6	45,2	15,0	0,636
Costuma comprar alimentos em feiras livres ou de rua	15,9	49,3	17,4	17,4	20,4	47,3	24,7	7,5	0,199
Costuma comer balas, chocolates e outras guloseimas	23,2	23,2	42,0	11,6	16,1	20,4	50,5	13,0	0,608
Costuma beber sucos industrializados	18,8	43,5	23,2	14,5	15,0	48,4	28,0	8,6	0,554
Costuma frequentar restaurantes fast-foods ou lanchonetes	7,2	63,8	27,5	1,5	2,2	50,5	41,9	5,4	0,055
Tem o hábito de beliscar entre as refeições	23,2	40,6	21,7	14,5	17,2	45,2	22,6	15,0	0,827
Costuma beber refrigerante	15,9	33,3	42,1	8,7	8,6	34,4	47,3	9,7	0,560
Costuma trocar comida por sanduíches, salgados, pizzas	17,4	50,7	27,5	4,4	3,2	61,3	31,2	4,3	0,021
Quando bebe café ou chá, costuma colocar açúcar	7,3	37,7	4,3	50,7	9,8	38,0	15,2	37,0	0,085

[a]Qui-quadrado e exato de Fisher

Tabela Suplementar 4. Prevalências (%) de práticas alimentares conforme escolaridade de usuários do serviço de nutrição na Atenção Primária. Pelotas, Rio Grande do Sul, 2022 (N=161)

	Ensino fundamental incompleto				Ensino fundamental completo				
**Domínio e Componente da escala**	**Nunca**	**Raramente**	**Muitas Vezes**	**Sempre**	**Nunca**	**Raramente**	**Muitas Vezes**	**Sempre**	**p-valor[a]**
	%	%	%	%	%	%	%	%	
Planejamento e organização doméstica									
Come frutas ou castanhas em pequenos lanches	18,8	28,7	29,7	22,8	33,3	26,7	23,3	16,7	0,219
Escolhe frutas, verduras e legumes de produção local	30,6	14,9	29,7	24,8	28,3	23,3	16,7	31,7	0,185
Leva algum alimento para caso sentir fome	7,9	73,3	7,9	10,9	5,0	65,0	13,3	16,7	0,410
Planeja refeições	26,7	37,6	13,9	21,8	16,7	63,3	6,7	13,3	0,021
Varia o consumo de feijão com outras leguminosas	27,7	27,7	30,7	13,9	13,3	40,0	40,0	6,7	0,047
É comum usar farinha integral	6,9	75,3	10,9	6,9	6,7	65,0	18,3	10,0	0,441
Costuma comer fruta no café da manhã	5,9	73,3	13,9	6,9	8,3	75,1	8,3	8,3	0,701
Modos de comer									
Costuma fazer as refeições sentado(a) à mesa	22,8	7,9	9,9	59,4	8,3	11,7	15,0	65,0	0,091
Realiza as refeições com calma	15,8	5,9	10,9	67,4	21,7	6,7	30,0	41,7	0,005
Costuma participar do preparo das refeições	16,8	11,9	6,9	64,4	18,3	6,7	11,7	63,3	0,557
Na casa as pessoas compartilham as tarefas que envolvem o preparo e consumo das refeições	18,8	30,7	7,9	42,6	20,0	26,7	13,3	40,0	0,707
Aproveita o horário das refeições para resolver outras coisas e acaba deixando de comer	9,9	73,3	10,9	5,9	23,3	60,0	11,7	5,0	0,133
Costuma fazer as refeições na mesa de trabalho ou estudo	6,9	90,1	2,0	1,0	10,0	83,3	5,0	1,7	0,517
Costuma fazer as refeições sentado(a) no sofá ou na cama	24,8	53,5	13,8	7,9	16,6	50,0	16,7	16,7	0,274
Costuma pular pelo menos uma das refeições principais	32,7	40,6	19,8	6,9	38,3	33,3	21,7	6,7	0,816
Escolhas alimentares									
Dá preferência pra frutas, verduras e legumes orgânicos	13,1	21,3	40,9	24,6	12,2	24,4	46,3	17,0	0,821
Costuma comprar alimentos em feiras livres ou de rua	14,9	52,5	23,7	8,9	25,0	41,7	18,3	15,0	0,192
Costuma comer balas, chocolates e outras guloseimas	18,8	23,7	47,5	10,0	20,0	18,3	45,0	16,7	0,578
Costuma beber sucos industrializados	16,8	46,5	23,8	12,9	16,7	45,0	30,0	8,3	0,755
Costuma frequentar restaurantes fast-foods ou lanchonetes	1,0	64,3	32,7	2,0	10,0	41,7	41,7	6,6	0,003
Tem o hábito de beliscar entre as refeições	18,8	43,5	22,8	14,9	21,7	43,3	20,0	15,0	0,963
Costuma beber refrigerante	10,9	36,6	45,5	7,0	13,3	28,4	45,0	13,3	0,445
Costuma trocar comida por sanduíches, salgados, pizzas	8,9	63,4	25,7	2,0	10,0	45,0	36,7	8,3	0,060
Quando bebe café ou chá, costuma colocar açúcar	8,0	40,0	8,0	44,0	8,3	35,0	15,0	41,7	0,565

[a]Qui-quadrado e exato de Fisher

Tabela Suplementar 5. Prevalências (%) de práticas alimentares conforme tabagismo de usuários do serviço de nutrição na Atenção Primária. Pelotas, Rio Grande do Sul, 2022 (N=161)

Domínio e Componente da escala	Não				Sim				
	Nunca	Raramente	Muitas Vezes	Sempre	Nunca	Raramente	Muitas Vezes	Sempre	p-valor[a]
	%	%	%	%	%	%	%	%	
Planejamento e organização doméstica									
Come frutas ou castanhas em pequenos lanches	24,6	29,1	26,9	19,4	22,3	25,9	25,9	25,9	0,906
Escolhe frutas, verduras e legumes de produção local	28,4	17,2	23,9	30,6	33,3	22,2	29,6	14,8	0,380
Leva algum alimento para caso sentir fome	6,7	69,4	11,2	12,7	7,4	74,1	3,7	14,8	0,734
Planeja refeições	20,9	47,0	12,7	19,4	33,3	44,4	7,4	14,8	0,569
Varia o consumo de feijão com outras leguminosas	23,1	32,1	33,6	11,2	22,2	29,6	37,1	11,1	0,990
É comum usar farinha integral	7,5	69,4	15,6	7,5	3,7	81,5	7,4	7,4	0,671
Costuma comer fruta no café da manhã	6,7	75,3	10,5	7,5	7,4	66,7	14,8	11,1	0,667
Modos de comer									
Costuma fazer as refeições sentado(a) à mesa	17,2	7,5	11,9	63,4	14,8	18,5	11,1	55,6	0,380
Realiza as refeições com calma	16,4	6,7	18,7	58,2	22,2	3,7	14,8	59,3	0,851
Costuma participar do preparo das refeições	17,2	11,2	8,2	63,4	14,8	3,7	11,1	70,4	0,665
Na casa as pessoas compartilham as tarefas que envolvem o preparo e consumo das refeições	22,4	28,4	9,0	40,3	7,4	33,3	14,8	44,4	0,270
Aproveita o horário das refeições para resolver outras coisas e acaba deixando de comer	14,2	68,7	12,9	5,2	14,8	66,7	11,1	7,4	0,959
Costuma fazer as refeições na mesa de trabalho ou estudo	8,2	87,3	3,0	1,5	7,4	92,6	0,0	0,0	1,000
Costuma fazer as refeições sentado(a) no sofá ou na cama	20,2	52,2	17,2	10,5	25,9	55,6	3,7	14,8	0,283
Costuma pular pelo menos uma das refeições principais	32,1	38,1	23,1	6,7	48,2	40,7	3,7	7,4	0,070
Escolhas alimentares									
Dá preferência pra frutas, verduras e legumes orgânicos	13,4	19,4	48,5	18,7	25,9	22,2	40,7	11,1	0,351
Costuma comprar alimentos em feiras livres ou de rua	17,9	46,3	23,1	12,7	22,2	59,3	11,1	7,4	0,409
Costuma comer balas, chocolates e outras guloseimas	19,4	16,4	50,0	14,2	18,5	48,2	29,6	3,7	0,004
Costuma beber sucos industrializados	15,7	46,3	26,1	11,9	18,5	48,2	25,9	7,4	0,936
Costuma frequentar restaurantes fast-foods ou lanchonetes	2,9	55,2	37,3	4,5	11,1	62,9	25,9	0,0	0,149
Tem o hábito de beliscar entre as refeições	17,9	44,0	21,6	16,4	29,6	40,7	25,9	3,7	0,212
Costuma beber refrigerante	9,0	32,8	47,0	11,2	22,2	40,8	37,0	0,0	0,052
Costuma trocar comida por sanduíches, salgados, pizzas	9,0	53,7	32,1	5,2	11,1	74,1	14,8	0,0	0,147
Quando bebe café ou chá, costuma colocar açúcar	10,5	37,6	12,0	39,9	0,0	40,7	3,7	55,6	0,136

[a]Qui-quadrado e exato de Fisher

Tabela Suplementar 6. Prevalências (%) de práticas alimentares conforme prática de atividade física no lazer de usuários do serviço de nutrição na Atenção Primária. Pelotas, Rio Grande do Sul, 2022 (N=161)

Domínio e Componente da escala	Não				Sim				
	Nunca	Raramente	Muitas Vezes	Sempre	Nunca	Raramente	Muitas Vezes	Sempre	p-valor[a]
	%	%	%	%	%	%	%	%	
Planejamento e organização doméstica									
Come frutas ou castanhas em pequenos lanches	25,7	28,6	29,5	16,2	21,4	28,6	21,4	28,6	0,281
Escolhe frutas, verduras e legumes de produção local	30,5	18,1	24,8	26,7	26,8	17,9	25,0	30,4	0,950
Leva algum alimento para caso sentir fome	7,6	70,5	8,6	13,3	5,4	69,6	12,5	12,5	0,845
Planeja refeições	20,0	51,4	12,4	16,2	28,6	37,5	10,7	23,2	0,286
Varia o consumo de feijão com outras leguminosas	21,0	35,2	35,2	8,6	26,8	25,0	32,1	16,1	0,295
É comum usar farinha integral	5,7	72,4	12,4	9,5	8,9	69,6	17,9	3,6	0,379
Costuma comer fruta no café da manhã	4,8	80,9	8,6	5,7	10,7	60,7	16,1	12,5	0,043
Modos de comer									
Costuma fazer as refeições sentado(a) à mesa	17,1	12,4	13,3	57,1	16,1	3,6	8,9	71,4	0,189
Realiza as refeições com calma	21,0	7,6	13,3	58,1	10,7	3,6	26,8	58,9	0,087
Costuma participar do preparo das refeições	18,1	11,4	6,7	63,8	14,3	7,1	12,5	66,1	0,505
Na casa as pessoas compartilham as tarefas que envolvem o preparo e consumo das refeições	18,1	31,4	10,5	40,0	23,2	25,0	8,9	42,9	0,762
Aproveita o horário das refeições para resolver outras coisas e acaba deixando de comer	16,2	67,6	11,4	4,8	10,7	69,6	12,5	7,1	0,742
Costuma fazer as refeições na mesa de trabalho ou estudo	2,9	94,3	2,9	0,0	17,9	76,8	1,8	3,6	0,001
Costuma fazer as refeições sentado(a) no sofá ou na cama	21,9	45,7	18,1	14,3	19,6	66,1	8,9	5,4	0,056
Costuma pular pelo menos uma das refeições principais	37,1	36,2	20,0	6,7	30,4	42,9	19,6	7,1	0,800
Escolhas alimentares									
Dá preferência pra frutas, verduras e legumes orgânicos	16,2	20,0	48,6	15,2	14,3	19,6	44,6	21,4	0,797
Costuma comprar alimentos em feiras livres ou de rua	21,9	45,7	21,9	10,5	12,5	53,6	19,6	14,3	0,436
Costuma comer balas, chocolates e outras guloseimas	22,8	20,0	45,7	11,4	12,5	25,0	48,2	14,3	0,425
Costuma beber sucos industrializados	13,3	42,9	29,5	14,3	21,4	53,6	19,6	5,4	0,095
Costuma frequentar restaurantes fast-foods ou lanchonetes	5,7	60,0	30,5	3,8	1,8	50,0	44,6	3,6	0,282
Tem o hábito de beliscar entre as refeições	20,0	40,0	23,8	16,2	19,6	50,0	19,6	10,7	0,621
Costuma beber refrigerante	15,2	26,7	46,7	11,4	3,6	48,2	42,8	5,4	0,012
Costuma trocar comida por sanduíches, salgados, pizzas	8,6	59,0	26,6	4,8	10,7	53,6	32,1	3,6	0,857
Quando bebe café ou chá, costuma colocar açúcar	8,7	31,7	12,5	47,1	9,0	50,0	7,1	33,9	0,137

[a]Qui-quadrado e exato de Fisher
