# Supplementary material for: Cross-sectional study of dietary practices from the Dietary Guidelines for the Brazilian Population among users of a Basic Health Unit in Pelotas, 2022
Source: Epidemiol Serv Saude. 2025 Aug 4;34:e20240420. doi: 10.1590/S2237-96222025v34e20240420.en (PMC12334227; doi:10.1590/S2237-96222025v34e20240420.en)
Supplement: Translation

# Translation: Estudo transversal das práticas alimentares do Guia Alimentar para a População Brasileira em usuários de uma Unidade Básica de Saúde de Pelotas, 2022

**DOI:** 10.1590/S2237-96222025v34e20240420.pt

**Authors:** Mabel Nilson Alves, Denise Petrucci Gigante, Gicele Costa Mintem, Francine Silva dos Santos

**Affiliations:** 1 Universidade Federal de Pelotas, Faculdade de Nutrição, Programa de Pós- Graduação Nutrição e Alimentos, Pelotas, RS, Brasil; 2 Universidade Federal de Ciências da Saúde de Porto Alegre, Departamento de Nutrição, Porto Alegre, RS, Brasil

**Keywords:** Dieta saudável, Atenção primária à saúde, Guias alimentares, Saúde pública, Estudos transversais

## Abstract

**Objetivo:**

Descrever práticas alimentares recomendadas no Guia Alimentar e a associação com fatores sociodemográficos e hábitos de vida em usuários do serviço de nutrição de uma Unidade Básica de Saúde em Pelotas, Rio Grande do Sul no ano de 2022.

**Métodos:**

Estudo transversal com indivíduos com idade ≥20 anos que receberam atendimento nutricional. O questionário utilizado incluiu uma escala validada para a população brasileira, que considera três domínios do consumo alimentar: planejamento e organização doméstica, escolha dos alimentos e modos de comer. As práticas que obtiveram frequência de 60% ou mais foram consideradas adequadas às recomendações do Guia Alimentar.

**Resultados:**

Entre os 162 entrevistados, somente as práticas alimentares relacionadas com o domínio dos modos de comer foram adequadas. A média do escore de adequação foi de 39,0 pontos com desvio padrão de 5,4 pontos e com mediana de 39,0 pontos. A média do escore de adequação foi estatisticamente mais elevada nos entrevistados que se autodeclararam de raça/cor da pele pardos/pretos, que apresentaram 40,4 pontos com intervalo de confiança de 95% (IC95% 39,1; 41,7) e entre os ativos no lazer, que apresentaram 40,4 pontos (IC95% 39,0; 41,8), após ajustes para variáveis demográficas e socioeconômicas.

**Conclusão:**

Os valores do escore de adequação foram maiores em indivíduos pardos/pretos e praticantes de atividade física no lazer. Práticas relacionadas aos domínios de planejamento e escolhas dos alimentos devem ser priorizadas no aconselhamento nutricional.

**Aspectos éticos**

A presente pesquisa respeitou os princípios éticos, obtendo os seguintes dados de aprovação: :

Comitê de ética em pesquisa: Universidade Federal de Pelotas

Número do parecer: 5.373.094

Data de aprovação: 27/4/2022

Certificado de apresentação de apreciação ética: 57491222.2.0000.5316

Registro do consentimento livre e esclarecido: Obtido de todos os participantes antes da coleta.

## Introdução

A Política Nacional de Atenção Básica compreende um vasto conjunto de ações de saúde devendo ser desenvolvida por meio de práticas de cuidado integrado, gestão qualificada e dirigida à população em território definido (1). Sob essa ótica, a Política Nacional de Alimentação e Nutrição tem como propósito a melhoria das condições de alimentação, nutrição e saúde da população brasileira, mediante a promoção de práticas alimentares adequadas e saudáveis, a vigilância alimentar e nutricional, a prevenção e o cuidado integral dos agravos relacionados à alimentação e nutrição (2). 

Para essas ações o Ministério da Saúde orienta os profissionais da Atenção Primária à saúde a utilizarem o *Guia Alimentar para a População Brasileira* (3). Em 2014, ele foi atualizado e incluiu a classificação NOVA, baseada na extensão e propósito do grau de processamento dos alimentos (3). Ampliou as recomendações, para além da dimensão biológica, ao considerar aspectos ambientais, econômicos, sociais, culturais e alimentação como um direito. É pioneiro em incentivar o desenvolvimento de habilidades culinárias, a transmissão do conhecimento culinário entre as gerações, o incentivo da divisão de tarefas na cozinha e o comer em família (4).

Em contraponto ao recomendado pelo Guia alimentar, se observa que em 16 anos, os brasileiros diminuíram em 7,0% o consumo de alimentos in natura, aumentaram em 46,0% o consumo de alimentos ultraprocessados e aumentaram em 250,0% a compra de refeições prontas congeladas (5). No mesmo período, cresceu a prevalência de doenças crônicas relacionadas à má alimentação (5). Na Atenção Primária, 37,0% dos indivíduos adultos referiram consumir hambúrgueres e/ou embutidos no dia anterior e 54,0% ingeriram bebidas adoçadas no mesmo período (6).

Dada a evidente importância das orientações do Guia Alimentar, mais investigações sobre a adesão às suas práticas recomendadas na Atenção Primária são necessárias. Estudar as práticas alimentares à luz do *Guia Alimentar para a População Brasileira* de indivíduos assistidos na Atenção Primária à saúde, especialmente os já atendidos por nutricionista, propicia melhor entendimento para a sua implementação nessa população, podendo atingir melhores desfechos em saúde (7-8). Sendo assim, o objetivo deste estudo foi descrever práticas alimentares recomendadas no *Guia Alimentar para a População Brasileira* e a associação com fatores sociodemográficos e hábitos de vida em usuários do serviço de nutrição de uma Unidade Básica de Saúde em Pelotas, Rio Grande do Sul, no ano de 2022.

## Métodos

### Delineamento do estudo 

Estudo transversal com seleção da população-alvo por conveniência.

### Contexto

O estudo foi realizado em uma Unidade Básica de Saúde localizada no bairro Três Vendas do município de Pelotas, Rio Grande do Sul. Pelotas apresenta 325.685 pessoas residentes e Índice de Desenvolvimento Humano Municipal de 0,739 (9). A Atenção Primária do município está organizada em Redes de Atenção à Saúde, que desempenham ações e serviços de saúde articulados de forma complementar e com base territorial, sendo as Unidades Básicas de Saúde o início das Redes. Atualmente, Pelotas conta com 50 Unidades Básicas de Saúde (10). Dessas, três Unidades estão vinculadas à Universidade Federal de Pelotas onde são desenvolvidas atividades de ensino, pesquisa e extensão (11). A Unidade Básica de Saúde, local do presente estudo, foi escolhida por conveniência entre essas, por ser local de trabalho de uma das autoras para diagnosticar a situação de adequação das práticas alimentares dos pacientes atendidos pelo serviço de nutrição. Essa Unidade possuía, no momento da coleta de dados, população adscrita de 3500 pessoas e apresenta modelo de atenção mista, ou seja, Unidade Básica de Saúde tradicional e Estratégia Saúde da Família. Na sua área adscrita apresenta apenas uma feira de alimentos in natura, que comercializa seus produtos em somente um dia por semana.

### Participantes

A população-alvo do presente estudo é constituída por indivíduos com 20 anos ou mais que receberam atendimento nutricional. A seleção dos indivíduos foi por meio de análise de todos os registros de pacientes atendidos pela equipe de nutrição da Unidade no período de 2015 a 2019. Como critério de inclusão acrescentou-se residir na área adscrita da Unidade Básica de Saúde, no momento do atendimento nutricional e da coleta dos dados para o estudo. Como critério de exclusão foi considerada alguma condição (física ou mental) que limitasse a resposta dos selecionados ao questionário. 

### Variáveis

Os desfechos no presente estudo foram as práticas alimentares adequadas às recomendações do Guia Alimentar para a População Brasileira. Para isso utilizou-se o escore de adequação da escala validada para a população brasileira, constituída por 24 questões e dividida em três domínios. No domínio planejamento e organização doméstica estão contidos os questionamentos sobre sete práticas; no domínio “modos de comer”, oito práticas e no domínio “escolhas alimentares”, nove práticas. Cada pergunta apresenta como opções de resposta nunca, raramente, muitas vezes e sempre. Para cada resposta há uma pontuação que, somadas, resulta no escore de adequação que varia da pontuação mínima de 0 até a máxima de 72 pontos, sendo considerado adequado o valor ≥42 pontos (12). Em acréscimo, utilizou-se para cada prática o parâmetro de frequência ≥60% para classificar como frequência adequada (13). No presente estudo, a escala foi modificada da forma afirmativa para interrogativa e da primeira para a terceira pessoa. 

As variáveis explicativas foram as demográficas, socioeconômicas e de estilo de vida. As demográficas analisadas foram: o sexo (feminino ou masculino), a idade em anos completos e analisada em duas categorias, adultos (20 a 59 anos) e idosos (≥60 anos), e a raça/cor da pele autodeclarada conforme as categorias estabelecidas em estudos nacionais (14) e, agrupadas posteriormente, em parda/negra ou branca. 

Como variáveis socioeconômicas: nível de escolaridade (ano e grau de estudo completado pelo entrevistado e convertida como variável quantitativa discreta em anos de estudo) e analisada na forma dicotômica (fundamental incompleto: até 7 anos de estudo e fundamental completo: 8 anos e mais) e renda familiar per capita, analisada em tercis, e obtida pela soma de todos os valores recebidos pelo participante e pelas pessoas residentes na mesma casa, dividida pelo número de moradores do domicílio.

Variáveis de estilo de vida: para atividade física utilizou-se a pergunta “O(a) senhor(a) faz algum esporte, exercício ou atividade física no lazer ou tempo livre?” com opções de resposta sim ou não, e analisada dessa forma. Para tabagismo foi considerado fumante o indivíduo que fuma independentemente do número de cigarros, da frequência e da duração do hábito de fumar (14). Inicialmente foi apresentado em três categorias: fumante, ex-fumante ou nunca fumou e nas análises bivariada e regressão linear a variável se apresenta de forma dicotômica em tabagismo “sim” e “não”, sendo essa última a categoria que incluiu não fumantes e ex-fumantes.

### Coleta de dados

A verificação da elegibilidade e a coleta de dados ocorreram de junho a novembro de 2022, por entrevistadoras treinadas (estudantes do curso de Nutrição). A coleta de dados foi realizada em prontuários físicos e eletrônicos e em entrevistas por chamada telefônica ou presenciais. 

O questionário foi construído no software Epicollect5. Como medidas de controle de qualidade dos dados, foi realizado um estudo piloto para adequar o questionário e a logística do estudo, as entrevistadoras foram previamente treinadas e receberam manual de instruções para o trabalho de campo. O manual continha orientações sobre o preenchimento correto do questionário e uso do software. Ainda, a equipe coordenadora do estudo foi composta por nutricionistas com ampla experiência na Atenção Primária da Saúde e na condução de estudos epidemiológicos. 

### Viés

O delineamento do presente estudo não possibilita estabelecer relação de causalidade, portanto faz a descrição e mostra associações entre os desfechos e as variáveis explicativas sem indicação de possíveis causas. Por se tratar de amostra de conveniência, apresenta generalização restrita, não sendo possível extrapolar os resultados para toda a população usuária da Atenção Primária.

### Tamanho do estudo

De 2015 a 2019, o número de atendimentos da nutrição foi de 2850, sendo de 1103 da área adscrita da Unidade. Desses, 380 eram indivíduos menores de 20 anos e 487 compareceram à Unidade Básica de Saúde apenas para aferição de medidas antropométricas do Programa Bolsa Família, não configurando atendimento nutricional. Então, 236 indivíduos eram elegíveis para o estudo. Após as buscas desses indivíduos identificaram-se: 15 mudanças de endereço, 14 óbitos e um indivíduo estava impossibilitado de responder ao questionário. Totalizando 206 indivíduos elegíveis para o estudo. Desses, 162 foram entrevistados (78,6%), as recusas representaram 13,1% e as perdas, 8,3%.

### Métodos estatísticos

Os dados obtidos na planilha eletrônica gerada pelo Epicollect5 foram importados pelo programa estatístico Stata 16.1 (Stata Corporation, College Station, Texas, Estados Unidos da América). Inicialmente, foram observadas as distribuições de frequência das variáveis dicotômicas, ordinais e categóricas quanto à normalidade da distribuição. Após, foi verificada a frequência de todas as variáveis de interesse, descrição das variáveis numéricas em médias e desvio-padrão e de proporção para as variáveis categóricas. O nível de significância estatística foi p-valor menor de 5,0%.

Foram calculadas as prevalências das variáveis e seus intervalos de confiança de 95% (IC95%). As análises bivariadas foram conduzidas entre os desfechos e as variáveis explicativas por meio dos testes estatísticos Qui-quadrado e exato de Fisher. Regressão linear foi conduzida para avaliar as médias dos escores de adequação em relação às variáveis explicativas utilizando o teste de Wald. Para as análises ajustadas utilizou-se um modelo hierarquizado em níveis, sendo o primeiro nível composto pelas variáveis demográficas (sexo, idade e raça/cor da pele); o segundo nível, pelas variáveis socioeconômicas (escolaridade e renda per capita) e o terceiro nível, pelas variáveis de hábitos de vida (tabagismo e atividade física). Estimativas foram ajustadas para variáveis ​​do mesmo nível e do nível anterior, consideradas como possíveis fatores de confusão, e permaneceram no modelo aquelas que apresentaram p-valor menor de 20,0% em seu nível hierárquico (15). 

## Resultados

Das 162 entrevistas realizadas, 57,4% ocorreram de forma presencial. As mulheres representaram 78,8%; pessoas com 40 anos ou mais, 83,3%; autodeclarados com raça/cor da pele branca, 57,4%; 62,7% referiram ter escolaridade inferior a oito anos e 65,2% não praticavam atividade física no lazer (Tabela 1).

**Tabela 1:** Distribuição absoluta e relativa dos usuários de um serviço de nutrição na Atenção Primária, segundo variáveis sociodemográficas e de estilo de vida. Pelotas, 2022 (n=162)

Variáveis	n (%)
Total	162 (100)
Sexo	
Masculino	36 (22,2)
Feminino	126 (77,8)
**Idade** (anos)^a^	
20-39	27 (16,7)
40-59	71 (43,8)
≥60	64 (39,5)
**Raça/cor da pele**	
Brancos	93 (57,4)
Pardos	23 (14,2)
Pretos	46 (28,4)
**Escolaridade** (anos)^a^	
0-4	100 (62,1)
5-7	1 (0,6)
8-10	11 (6,8)
11 ou mais	49 (30,5)
**Renda per capita** (reais)^b^	
1º tercil (0-454,5)	54 (33,8)
2º tercil (442,5-954,0)	53 (33,1)
3º tercil (954,1-4.893,3)	53 (33,1)
Tabagismo^a^	
Nunca fumou	87 (54,0)
Ex-fumante	47 (29,2)
Fumante	27 (16,8)
Atividade física no lazera	
Não	105 (65,2)
Sim	56 (34,8)

^a^n=161; ^b^n=160

Os indivíduos não apresentaram adequação a nenhuma prática do domínio planejamento e organização doméstica. No domínio “modos de comer” houve adequação em sete das oito práticas com amplitude de frequências de adequação de 72,8% a 95,7%. A prática de maior adequação nesse domínio foi nunca ou raramente fazer as refeições na mesa de trabalho ou estudo. No domínio “escolhas alimentares” houve adequação em cinco das nove práticas relacionadas com amplitude de frequência de adequação de 60,5% a 66,0%. A prática de maior adequação nesse domínio foi de nunca ou raramente trocar comida por sanduíches, salgados, pizzas (Tabela 2).

**Tabela 2:** Frequências e intervalos de confiança de 95% (IC95%) das práticas alimentares de usuários do serviço de nutrição na Atenção Primária. Pelotas, 2022 (n=162)

Domínio e componente da escala	Nunca/raramente	Muitas vezes/sempre
	% (IC95%)	% (IC95%)
**Planejamento e organização doméstica**		
Come frutas ou castanhas em pequenos lanches	52,5 (45,0; 60,1)	47,5 (40,0; 55,3)
Escolhe frutas, verduras e legumes de produção local	47,5 (39,9; 55,3)	52,5 (44,7; 60,1)
Leva algum alimento para caso sentir fome	76,5 (69,4; 82,5)	23,5 (17,5; 30,7)
Planeja refeições	69,7 (62,2; 76,4)	30,5 (23,6; 37,8)
Varia o consumo de feijão com outras leguminosas	54,9 (47,2; 62,5)	45,1 (37,5; 52,8)
É comum usar farinha integral	77,8 (70,7; 83,6)	22,2 (16,4; 29,3)
Costuma comer fruta no café da manhã	80,2 (73,3; 85,7)	19,8 (14,3; 26,7)
**Modos de comer**		
Costuma fazer as refeições sentado(a) à mesa	26,5 (20,3; 33,9)	73,5 (66,1; 79,7)
Realiza as refeições com calma	24,1 (18,1; 31,3)	75,9 (68,7; 81,9)
Costuma participar do preparo das refeições	27,2 (20,8; 34,6)	72,8 (65,4; 79,2)
Na casa as pessoas compartilham as tarefas que envolvem o preparo e consumo das refeições	48,8 (41,1; 56,5)	51,2 (43,5; 58,9)
Aproveita o horário das refeições para resolver outras coisas e acaba deixando de comer	82,7 (76,5; 87,8)	17,3 (12,2; 23,9)
Costuma fazer as refeições na mesa de trabalho ou estudo	95,7 (91,2; 97,9)	4,3 (2,1; 8,8)
Costuma fazer as refeições sentado(a) no sofá ou na cama	74,1 (66,7; 80,3)	25,9 (19,7; 33,3)
Costuma pular pelo menos uma das refeições principais	72,8 (65,4; 79,2)	27,2 (20,8; 34,6)
**Escolhas alimentares**		
Dá preferência para frutas, verduras e legumes orgânicos	35,8 (28,8; 43,5)	64,2 (56,5; 71,3)
Costuma comprar alimentos em feiras livres ou de rua	66,7 (59,0; 73;5)	33,3 (26,5; 41,0)
Costuma comer balas, chocolates e outras guloseimas	40,7 (33,4; 48,5)	59,3 (51,5; 66,6)
Costuma beber sucos industrializados	62,9 (55,2; 70,1)	37,0 (29,9; 44,8)
Costuma frequentar restaurantes fast-foods ou lanchonetes	60,5 (52,7; 67,8)	39,5 (32,2; 47,3)
Tem o hábito de beliscar entre as refeições	63,0 (55,2; 70,1)	37,0 (29,9; 44,8)
Costuma beber refrigerante	45,7 (38,1; 53,5)	54,3 (46,6; 61,9)
Costuma trocar comida por sanduíches, salgados, pizzas	66,0 (58,4; 73,0)	33,9 (27,0; 41,6)
Quando bebe café ou chá, costuma colocar açúcar	46,6 (39,0; 54,4)	53,4 (45,6; 61,0)

Indivíduos pardos/pretos, comparados aos brancos, referiram com maior frequência muitas vezes e sempre escolher frutas, verduras de produção local (59,4% vs. 47,3%; p-valor 0,003); sempre comer fruta no café da manhã (13,0% vs. 4,3%; p-valor 0,036); nunca aproveitar o horário das refeições para resolver outras coisas e acabar deixando de comer (21,5 vs. 9,7%; p-valor 0,002); nunca fazer as refeições no sofá ou na cama (29,0% vs. 16,1%; p-valor 0,037) e nunca trocar a comida do almoço ou jantar por sanduíches, salgados ou pizza (17,4% vs. 3,2%; p-valor 0,021). Indivíduos pardos/pretos, comparados aos brancos, referiram com menor frequência sempre pular refeições principais (almoço ou jantar) (4,4% vs. 8,6%; p-valor 0,049) (Tabela Suplementar 3). Indivíduos praticantes de atividade física, comparados aos não praticantes, relataram com maior frequência sempre comer fruta no café da manhã (12,5% vs. 5,7%; p-valor 0,043) e com menor frequência sempre beber refrigerante (5,4% vs. 11,4%; p-valor 0,012) (Tabela Suplementar 6). Associações das práticas alimentares com outras variáveis explicativas como sexo, grupo etário, escolaridade e tabagismo são apresentadas nas Tabelas suplementares 1, 2, 4 e 5.

O escore de adequação variou de 28 a 53 pontos, com média de 39,0 pontos, desvio padrão de 5,4 pontos e mediana de 39,0 pontos. A média foi estatisticamente mais elevada nos entrevistados que se autodeclararam pardos/pretos sendo de 40,4 pontos com intervalo de confiança de 95% (IC95% 39,1; 41,7) e entre os ativos no lazer, que apresentaram 40,4 pontos (IC95% 39,0; 41,8), mesmo após os ajustes para as variáveis incluídas nos primeiro e segundo níveis do modelo de análise. Os entrevistados autodeclarados pardos/pretos apresentaram média de 2,44 pontos, maior do que a média do escore dos entrevistados brancos. Os praticantes de atividade física apresentaram 2,00 pontos a mais na média, em relação aos não praticantes (Tabela 3). 

**Tabela 3:** Médias e intervalo de confiança de 95% (IC95%) não ajustadas e ajustadas do escore de adequação pelas variáveis do estudo em usuários do serviço de nutrição na Atenção Primária. Pelotas, 2022 (n=162)

Variáveis	Não ajustada (IC95%)	p-valor^a^	Ajustada (IC95%)	p-valor^a^
**1º nível: variáveis demográficas** (n=162)				
Sexo		0,601		0,872
Feminino	39,2 (38,2; 40,1)		39,0 (38,0; 40,0)	
Masculino	38,6 (36,8; 40,4)		39,2 (37,3; 41,0)	
**Idade em anos completos**		0,746		0,498
Adultos	38,9 (37,8; 40,0)		38,8 (37,7; 39,9)	
Idosos	39,2 (37,9; 40,6)		39,1 (38,1; 40,7)	
**Raça/cor da pele**		0,007		0,007
Branca	38,0 (37,0; 39,1)		38,0 (36,9; 39,1)	
Parda/negra	40,4 (39,1; 41,6)		40,4 (39,1; 41,7)	
**2º nível: variáveis socioeconômicas** (n=161/160)				
Escolaridade		0,335		0,404
Fundamental incompleto	39,3 (38,2; 40,4)		39,3 (38,2; 40,4)	
Fundamental completo	38,5 (37,1; 39,8)		38,6 (37,2; 39,9)	
**Renda per capita**		0,325		0,256
1º tercil	39,9 (38,4; 41,3)		39,8 (38,3; 41,2)	
2º tercil	38,3 (36,8; 39,8)		38,1 (36,6; 39,5)	
3º tercil	39,0 (37,6; 40,5)		39,2 (37,8; 40,7)	
**3º nível: variáveis comportamentais** (n=161)				
Tabagismo		0,255		0,379
Não	38,8 (37,9; 39,8)		38,9 (38,0; 39,8)	
Sim	40,1 (38,1; 42,2)		39,9 (37,9; 41,9)	
**Atividade física**		0,021		0,023
Não	38,3 (37,3; 39,4)		38,4 (37,3; 39,4)	
Sim	40,4 (39,0; 41,8)		40,4 (39,0; 41,8)	

^a^Teste de Wald; o ajuste foi realizado para variáveis do mesmo nível e do nível anterior com p-valor<0.20.

## Discussão

Os achados do presente estudo mostram que indivíduos adultos e idosos que receberam atendimento nutricional em uma unidade básica de saúde não apresentaram adequação a qualquer uma das práticas alimentares recomendadas no *Guia Alimentar para a População Brasileira* para o domínio planejamento e organização doméstica, já no domínio “modos de comer” houve adequação na maioria das práticas e em cinco das nove práticas do domínio de “escolhas alimentares”. Além disso, o escore médio da escala não alcançou a pontuação considerada para adequação (≥42 pontos) (12), sendo maior entre os entrevistados que se autodeclararam pardos/pretos em relação aos brancos e nos praticantes de atividade física comparados com os não praticantes. 

A não adequação das práticas relacionadas ao planejamento e organização doméstica pode ser reflexo da dificuldade de entendimento das práticas desse domínio, haja vista que o percentual de indivíduos com ensino fundamental incompleto identificado no estudo foi maior que a prevalência nacional (16). Soma-se a isso, a situação financeira dos indivíduos, que apresentaram renda mensal per capita bastante inferior àquela identificada em nível nacional e regional no mesmo ano (17). 

Sobre as escolhas alimentares incidem os fatores econômicos adicionados dos sociais e políticos que condicionam a situação alimentar da população, comunidades e indivíduos (18). Fato evidenciado na população estudada, uma vez que apresentou maior prevalência em relação a nunca ou raramente comprar alimentos em feiras livres, maior prevalência de muitas vezes ou sempre consumir guloseimas e refrigerantes. Essa relação corrobora com o aumento no preço dos alimentos in natura ou minimamente processados e redução do preço dos alimentos ultraprocessados ocorrido nas últimas três décadas (19). Outro fator diz respeito a que 80,0% das feiras de rua na cidade de Pelotas, Rio Grande do Sul concentrarem-se na região central, com poucas opções para os moradores dos bairros, onde residem os indivíduos do presente estudo (20). Os resultados do presente estudo relacionados com a menor adequação nos domínios de planejamento e organização doméstica e escolhas alimentares evidenciam a necessidade de medidas que visem assegurar um sistema alimentar saudável e sustentável de forma a contribuir para a melhoria de práticas alimentares relacionadas com esses domínios (21). O trabalho intensificado das equipes da Atenção Primária por meio de campanhas de orientação sobre as práticas saudáveis de alimentação aliado às ações do governo municipal para aumentar o número de feiras da agricultura familiar, com aumento na demanda por esses produtos, poderá promover maior diversidade e acesso a alimentos saudáveis. Ainda assim, são necessários subsídios financeiros governamentais para modular o sistema alimentar a fim de promover a alimentação adequada e saudável entre os indivíduos estudados. 

Já as práticas do domínio modos de comer apresentaram maiores prevalências de adequação às recomendações do Guia Alimentar. Torna-se notório que elas se mostram relacionadas ao comportamento alimentar e, portanto, não dependem de condições financeiras para sua realização. Além disso, representam hábitos ainda mantidos por esses indivíduos, exemplificando um patrimônio imaterial brasileiro que necessita ser preservado e perpetuado (22). A equipe de Atenção Primária apresenta o potencial de divulgar e encorajar essas práticas por meio da estratégia saúde da família (1).

A média e a mediana do escore de adequação encontram-se abaixo do ponto de corte considerado para aqueles que apresentam práticas alimentares adequadas e saudáveis (≥42 pontos) (12), reforçando que essas práticas precisam melhorar nessa população. No entanto, foi ainda superior ao identificado em trabalhadores do comércio de serviços de alimentação (lanchonetes, restaurantes, padarias, cafeterias, super e hipermercados) de Fortaleza, Ceará, cujo escore de adequação médio foi 35 pontos (23) e maior do que o escore de adequação médio de 36 pontos encontrados em estudo realizado com 900 adultos de todo o Brasil. (9). A maior média do escore de adequação encontrada no presente estudo em relação aos outros, relatados anteriormente, pode ser fruto do tratamento nutricional recebido na Unidade Básica de Saúde, exemplificando que intervenções nutricionais podem influenciar na qualidade da alimentação mesmo em indivíduos de baixa renda (24). 

Indivíduos que se autodeclararam pardos/pretos apresentaram as maiores médias do escore de adequação das práticas alimentares adequadas às recomendações do *Guia Alimentar para a População Brasileira*, o que está relacionado aos relatos a respeito de muitas vezes ou sempre escolher frutas e verduras da produção local, nunca ou raramente pular refeições e nunca trocar a comida do almoço ou jantar por sanduíches, salgados ou pizza. Esses achados podem ser devido à alimentação tradicional brasileira ainda preservada entre os indivíduos pardos/pretos (25). Em nível nacional foi identificado maior consumo de alimentos in natura e minimamente processados, especialmente arroz e feijão, entre os indivíduos pardos/pretos (26).

A média no escore de adequação das práticas alimentares adequadas às recomendações do *Guia Alimentar para a População Brasileira* também foi mais elevada entre os praticantes de atividade física no lazer, o que está relacionado a maior frequência de sempre consumirem frutas no café da manhã e nunca ou raramente consumirem refrigerantes. Isso está de acordo com o Modelo de Crenças em Saúde, o qual sugere que os indivíduos tendem a fazer um conjunto de escolhas saudáveis em seu estilo de vida na prevenção/tratamento de doenças quando estão conscientes que suas atitudes têm impacto na saúde (27). 

As limitações do estudo correspondem às perdas e recusas que representaram 21,0% dos indivíduos elegíveis. Para minimizar as perdas, quando o contato telefônico não era efetivado em pelo menos três tentativas em turnos diferentes, buscas presenciais eram realizadas nos domicílios em quatro tentativas em dias e turnos diferentes. O contato com os vizinhos para verificar a possibilidade de mudança de endereço ou falecimento também foi realizado. O cadastro nacional de falecidos também foi consultado. Por outro lado, o número de perdas pode ter sido superestimado haja vista que não foi possível confirmar se todas as perdas eram de fato de indivíduos elegíveis para o estudo, ou seja, moradores da área de abrangência da Unidade Básica de Saúde.

O presente estudo avança em apresentar a frequência das práticas alimentares recomendadas pelo *Guia Alimentar para a População Brasileira* por indivíduos atendidos na Atenção Primária, que já haviam recebido tratamento nutricional. Vale ressaltar, que o instrumento utilizado para avaliar o desfecho também conta como diferencial deste estudo, pois além de ser uma escala validada para a população brasileira (28), é instrumento oficial de avaliação da alimentação inserida no aplicativo Meu SUS Digital e no fôlder do Ministério da Saúde para avaliar a adesão às recomendações do *Guia Alimentar para a População Brasileira* (29). Ainda, como contribuição científica, o presente estudo analisou cada prática alimentar em relação às variáveis sexo, idade, raça/cor da pele, escolaridade, renda per capita, tabagismo e prática de atividade física, o que amplia a compreensão para o planejamento de ações voltadas para a melhoria da alimentação de indivíduos da Atenção Primária. 

Portanto, no presente estudo a média do escore de adequação às práticas recomendadas pelo Guia Alimentar foi abaixo do valor recomendado, sendo que os indivíduos que se autodeclararam pardos/pretos e os praticantes de atividade física mostraram médias mais elevadas em relação aos que se autodeclararam brancos e não praticantes de atividade física. Os indivíduos apresentaram maiores problemas de adequação de práticas alimentares relacionadas com as dimensões de planejamento e organização doméstica e escolha dos alimentos. Sugere-se o trabalho continuado e intensificado dos profissionais da Atenção Primária à saúde em sinergia com ações governamentais para divulgação, incentivo e apoio às práticas alimentares saudáveis recomendadas pelo *Guia Alimentar para a População Brasileira*.

## Data Availability

O banco de dados e os códigos de análise utilizados na pesquisa estão disponíveis mediante solicitação aos autores.
